# Association of Arsenic Methylation Capacity with Developmental Delays and Health Status in Children: A Prospective Case–Control Trial

**DOI:** 10.1038/srep37287

**Published:** 2016-11-17

**Authors:** Yu-Mei Hsueh, Wei-Jen Chen, Chih-Ying Lee, Ssu-Ning Chien, Horng-Sheng Shiue, Shiau-Rung Huang, Ming-I Lin, Shu-Chi Mu, Ru-Lan Hsieh

**Affiliations:** 1 Department of Family Medicine, Taipei Medical University Hospital, Taipei, Taiwan; 2 Department of Public Health, School of Medicine, College of Medicine, Taipei Medical University, Taipei, Taiwan; 3 School of Public Health, College of Public Health and Nutrition, Taipei Medical University, Taipei, Taiwan; 4 School of Medicine, College of Medicine, Taipei Medical University, Taipei, Taiwan; 5 Department of Chinese Medicine, Chang Gung Memorial Hospital and Chang Gung University College of Medicine, Taoyuan, Taiwan; 6 Department of Pediatrics, Shin Kong Wu Ho-Su Memorial Hospital, Taipei, Taiwan; 7 Department of Physical Medicine and Rehabilitation, Shin Kong Wu Ho-Su Memorial Hospital, Taipei, Taiwan; 8 Department of Physical Medicine and Rehabilitation, School of Medicine, College of Medicine, Taipei Medical University, Taipei, Taiwan

## Abstract

This case–control study identified the association between the arsenic methylation capacity and developmental delays and explored the association of this capacity with the health status of children. We recruited 120 children with developmental delays and 120 age- and sex-matched children without developmental delays. The health status of the children was assessed using the Pediatric Quality of Life Inventory (PedsQL) and Pediatric Outcomes Data Collection Instrument (PODCI). The arsenic methylation capacity was determined by the percentages of inorganic arsenic (InAs%), monomethylarsonic acid (MMA^V^%), and dimethylarsinic acid (DMA^V^%) through liquid chromatography and hydride generation atomic absorption spectrometry. Developmental delays were significantly positively associated with the total urinary arsenic concentration, InAs%, and MMA^V^%, and was significantly negatively associated with DMA^V^% in a dose-dependent manner. MMA^V^% was negatively associated with the health-related quality of life (HRQOL; −1.19 to −1.46, *P* < 0.01) and functional performance (−0.82 to −1.14, *P* < 0.01), whereas DMA^V^% was positively associated with HRQOL (0.33–0.35, *P* < 0.05) and functional performance (0.21–0.39, *P* < 0.01–0.05) in all children and in those with developmental delays. The arsenic methylation capacity is dose-dependently associated with developmental delays and with the health status of children, particularly those with developmental delays.

Exposure to environmental toxicants can affect health, particularly that of foetuses, infants, and children, during the sensitive and vulnerable early developmental and growth stages. Among heavy metals, arsenic is considered the most hazardous and toxic contaminant originating from environmental, medicinal, and occupational sources^1^,^2^, such as contaminated drinking water, silicon-based computer chips, pesticides, and feed additives for swine and poultry^3^. Arsenic toxicity depends on the total arsenic concentration and its oxidation state and chemical form^4^. InAs is methylated to MMA^V^ and DMA^V^, which are subsequently converted in arsenic metabolism to monomethylarsonous acid (MMA^III^) and dimethylarsinous acid (DMA^III^), respectively, and excreted in the urine in addition to unmetabolised InAs, namely arsenate (As^V^) and arsenite (As^III^)^2^. In most populations, 85% of InAs is methylated to at least MMA and mostly to DMA^5^. The arsenic methylation process is considered a detoxification process because MMA^V^ and DMA^V^ are less toxic than is InAs^6^. Arsenic exposure is associated with lung, liver, kidney, bladder, prostate, and skin cancers^7^. Morphological alterations subsequent to arsenic exposure during rapid brain growth periods evidenced arsenic-induced developmental toxicity^8^. Moreover, in utero exposure to arsenic can induce epigenetic changes and increase the risk of diseases in later stages of life^7^. The epidemiological burden caused by arsenic-induced environmental toxicity exerted negative effects on the growth and development of children and is a serious concern worldwide^9^.

Neurotrophins, including nerve growth factor (NGF), brain-derived neurotrophic factor (BDNF), neurotrophin-3, and neurotrophin-4, which act on p75NTR, TrkA, TrKB, and TrkC receptors, regulate various neurodevelopmental functions^10^. Neurotrophin-mediated endocytic trafficking regulates various developmental events, such as synaptogenesis, axonal growth, and even degenerative changes^11^. A recent molecular study reported that precursor and mature BDNF inhibit Schwann cell migration^12^. The nervous system maintains the most appropriate connections through competition among neurons for limiting the amount of neurotrophins produced by target cells^13^. Defective neurotrophin functions in the brain caused by a combination of environmental and genetic factors may result in many neurological diseases^14^. Inhibitory effects of recombinant human neurotrophin on arsenic trioxide-induced neurotoxicity were reported^15^.

Children with developmental delays have a lower health status, including HRQOL and functional performance, than do those with typical development^16^,^17^. Although with a relatively small sample size, we previously reported that higher urinary total arsenic levels and a lower arsenic methylation capacity with a higher MMA^V^% and lower DMA^V^% are associated with developmental delays in preschool children in a dose–response manner^16^. However, no study has established evidence regarding the arsenic methylation capacity and health status of children. Therefore, the present study evaluated the association between the arsenic methylation capacity and developmental delays in a larger sample and explored the association between this capacity and the health status of children.

## Results

The sociodemographic characteristics of the children and their mothers are shown in Table 1. The diagnosed developmental delays comprised cognitive dysfunctions (n = 5), speech–language delays (n = 14), gross and fine motor delays (n = 13), social and emotional delays (n = 12), global delays (n = 65), and unclassified delays (n = 11). Furthermore, we observed unclassified developmental disorders (n = 35), attention deficit hyperactivity disorder (n = 37), mental retardation (n = 14), autistic spectrum disorder (n = 10), articulation disorder or stuttering (n = 9), cerebral palsy (n = 7), and central nervous system-related disorders (n = 8). The mothers of the children with developmental delays had a significantly lower educational level than did those of the children without delays.

Table 2 presents the parent-reported health status and family functioning of the children. The children with developmental delays showed a significantly lower HRQOL and poorer functional performance than did those without developmental delays. Compared with the parents of the children without developmental delays, those of the children with developmental delays reported a significantly lower HRQOL, higher family influence, and increased psychological distress. We performed stratified analyses of urinary arsenic indices according to the age and sex of the children and educational level of their mothers. The results revealed that these indices were not associated with the age and sex of the children. Moreover, the children of mothers with a higher educational level exhibited a significantly higher DMA^V^% (91.75 ± 0.72 vs. 88.89 ± 0.90, *P* < 0.05), whereas those of mothers with a lower educational level had a significantly higher MMA^V^% (4.47 ± 0.48 vs. 2.91 ± 0.44, *P* < 0.05; data not shown).

Table 3 shows the dose–response association between the urinary arsenic indices and developmental delays. After adjustment for multiple variables, namely age, sex, educational level of the mothers, and urine creatinine, trend analyses of exposure strata in tertiles revealed the following dose–response associations. The association of developmental delays with the total urinary arsenic concentration, InAs%, and MMA^V^% was significantly positive, whereas that with DMA^V^% was significantly negative. These findings are consistent with our previous study regarding the association between the arsenic methylation capacity and developmental delays^16^, although the sample size was larger in this study.

Table 4 presents the association of the arsenic methylation capacity with the health status, namely the HRQOL and functional performance, of the participants determined using multiple linear regression analyses after adjustment for age, sex, and educational level of the mothers, according to the developmental status of the children. MMA^V^% showed a significantly negative association with the HRQOL (−1.19 to −1.46, *P* < 0.01) and functional performance (−0.82 to −1.14, *P* < 0.01), whereas DMA^V^% showed a significantly positive association with the HRQOL (0.33–0.35, *P* < 0.05) and functional performance (0.21–0.39, *P* < 0.01–0.05) in all children; the children with developmental delays demonstrated a similar tendency. However, in children without developmental delays, the association among MMA^V^%, psychosocial health, and global functioning was significantly negative and that among DMA^V^%, HRQOL, and functional performance was absent. In both groups, the total urinary arsenic concentration and InAs% were not associated with the health status, namely the HRQOL and functional performance (data not shown).

## Discussion

The present study provides further evidence regarding the dose–response association between poor arsenic methylation capacity and developmental delays. The health status, namely the HRQOL and functional performance, was negatively associated with MMA^V^% and positively associated with DMA^V^% in all children and in those with developmental delays. According to our review of relevant literature, this study is the first to reveal that poor arsenic methylation capacity (higher MMA^V^% and lower DMA^V^%) is negatively associated with the health status of children, particularly those with developmental delays.

In Taiwan, humans may be exposed to arsenic through drinking water, edible oil, cereals, rice, and seafood^16^. Approximately 70% of organic and inorganic arsenic is excreted through the kidney via urine^7^, and a small portion is excreted through the skin, hair, nails, and faeces^18^. Therefore, urinary arsenic speciation profiles are crucial for investigating the metabolism, toxicity, and transformation of ingested arsenic^19^,^20^, and they can be used as effective biomarkers for internal dosing^3^. By contrast, total arsenic in blood is an imprecise biomarker of exposure because of the relative toxicity and methylation efficiency of the different arsenic forms and the short-term effect of the exposure measure compared with total lead in blood^21^. Therefore, we used urinary arsenic species rather than blood arsenic levels to identify the association of the arsenic methylation capacity with developmental delays and the health status of the children in this study.

The primary metabolic pathway of InAs facilitates arsenic elimination through its methylation to MMA and DMA^22^. Physiological S-adenosylmethionine (SAM) is necessary for the methylation of InAs to MMA and subsequently to DMA^23^, and both methylation steps are catalysed by arsenic (+3 oxidation state) methyltransferase (AS3MT)^22^. SAM is biosynthesised through folate- and cobalamin-dependent one-carbon metabolism^22^. The arsenic methylation capacity is regulated by various enzymes and arsenic metabolism-related genes, such as AS3MT, purine nucleoside phosphorylase, and glutathione S-transferase omega 2^24^. Gene polymorphism in the one-carbon metabolism pathway through plasma folate and homocysteine metabolism may further lead to the risk of cancer^25^. Future research must identify the association between arsenic metabolism-related genes and developmental delays in children.

Various factors affect arsenic methylation in arsenic-exposed humans, including age, gender, race, lifestyle, inherited genetic characteristics, socioeconomic status, smoking, drinking, exposure route, arsenic species, and dietary factors such as fish, jaggery, tea, fruit, vitamins, N-acetylcysteine, glutathione, and zinc^6^,^9^,^26^,^27^. The arsenic methylation capacity was higher in children having a higher consumption of folate, meat, eggs, red–orange vegetables, and green leafy vegetables and higher body mass index^9^. For urinary arsenic species measurement, the participants were suggested to not consume seafood for at least 3 days before urine collection because of increases in DMA^V^ metabolism^28^. However, recent studies have reported that individual arsenic methylation patterns remained fairly stable for 5 days following arsenic ingestion^29^,^30^. We evaluated the association of the arsenic methylation capacity with development delays and the health status rather than the factors associated with the effects of the arsenic metabolism capacity on urinary arsenic excretion in children. Therefore, in this study, we neither assessed the factors related to arsenic metabolism, including neurotrophins and dietary state, nor restricted the diet and nutrition of the children during the intervention period. We could not determine the exact major exposure sources of arsenic in children; however, the urinary arsenic indices measured here can account for exposure to all sources^16^,^31^. The association between arsenic metabolism genes and poor arsenic methylation capacity and their associations with developmental delays warrant further investigation.

Studies have reported that arsenic can cross the placental barrier^32^,^33^,^34^. We could not rule out the possibility that developmental delays are caused by the arsenic exposure of the children rather than that of the mothers during pregnancy. Therefore, we further analysed the urinary arsenic methylation profiles of the mothers. These profiles showed no statistically significant differences in total urinary arsenic (15.02 ± 1.85 μg/L vs. 16.67 ± 2.28 μg/L, *P* = 0.58), InAs% (7.46 ± 0. 85 vs. 5.92 ± 0.99, *P* = 0.25), MMA^V^% (5.11 ± 0.94 vs. 4.36 ± 1.32, *P* = 0.63), and DMA^V^% (87.44 ± 1.42 vs. 89.72 ± 1.66, *P* = 0.30) among the mothers of the children with and without developmental delays. The results favour the speculation that developmental delays are caused by the arsenic exposure of the children rather than that of their mothers during pregnancy. However, because of the cross-sectional study design, additional longitudinal studies are required to confirm the speculation.

Although both MMA and DMA are methylated via similar pathways, MMA is considered more toxic than the other metabolites^35^. Decreased arsenic methylation capacity with high MMA% in urine is related to an increased cancer risk, noncancer diseases, and developmental delays^16^,^35^. We demonstrated positive correlations between DMA^V^ % and the health status, namely the HRQOL and functional performance, and negative correlations between MMA^V^ % and the health status. A possible explanation for these results is that a lower arsenic methylation capacity with lower MMA^V^% and higher DMA^V^% is either genetically related or influenced by environmental factors, such as long-term dietary and nutritional patterns^29^, which may be associated with the developmental status of the children; it further affects their health status, including the HRQOL. Because of the cross-sectional study design, future follow-up studies for children enrolled for a certain amount of time are indicated to determine whether the developmental delays can be alleviated and whether the HRQOL can improve with therapeutic interventions, such as changing their dietary habits and increasing the vitamin dosage.

The association of the arsenic methylation capacity with developmental disabilities evidenced by a deteriorated health status of the children was not as significant as that of the arsenic methylation capacity with developmental delays. Poor arsenic methylation capacity may contribute to developmental delays in children through various mechanisms. Furthermore, because of the case–control design with an exposure–outcome timeline constraint, we can claim only the presence of an association of arsenic exposure with developmental disabilities and could not differentiate this association from that of arsenic exposure with developmental delays in the present study. In addition, developmental delays themselves, rather than poor arsenic methylation capacity, may account for developmental disabilities in children. Therefore, the association between the arsenic methylation capacity and developmental disabilities in children may be caused by developmental delays. Additional longitudinal studies must explore the association of arsenic exposure with developmental delays and disabilities in children.

Arsenic exposure can cause developmental neurotoxicity in rat brains^36^. The oral administration of arsenic resulted in neurochemical and behavioural alterations in an animal model^37^. The exposure of pregnant rats to low InAs concentrations through drinking water during gestation and lactation can result in the delayed development of sensory–motor reflexes in rat pups and decreased development of locomotor activity in adult rats^38^. Arsenic exposure affects learning and concentration because arsenic can invade the blood–brain barrier^39^. A prospective cohort study conducted in Bangladesh reported that environmental arsenic exposure in early life hinders growth by the age of 5 years^40^. In a US pregnancy cohort study, low-level in utero arsenic exposure during gestation impaired the foetal immune regulation system, potentially causing disease in the future^41^. In rural Bangladesh, high arsenic exposure reduced the cell-mediated immunity in preschool children^42^. Concurrent arsenic exposure-induced immunosuppression may contribute to the risk of chronic diseases or infections^42^. Furthermore, in paediatric populations, an inverse association existed between InAs concentrations and judgment, attention, executive function, language, memory, visuospatial function, and processing speed^43^. We previously demonstrated a dose–response association between arsenic exposure and developmental delays^16^. However, the overall evidence did not reveal a specific causal dose–response relationship between arsenic exposure and cognitive function^21^. The differences in lifetime arsenic exposure and genetic polymorphisms among various populations may contribute to the varying results.

This study has several limitations. First, as mentioned, we could determine only associations between poor arsenic methylation capacity and developmental delays and between the arsenic methylation capacity and the health status of the children; this crucial point may affect the validity of our data interpretation. Second, because urinary arsenic concentrations were measured at a single time point, the exact sources of arsenic exposure for children remain unknown. However, the accuracy of urinary arsenic index evaluation by using spot urine samples might be reliable because all participants maintained their lifestyle and appeared to retain their homeostatic metabolism during the intervention period^16^. Third, the diagnoses of the children with developmental delays varied; therefore, we cannot extrapolate these results to a specific developmental delay in children, such as attention deficit hyperactivity disorder. However, the inclusion of participants with diverse diagnoses of developmental delays was advantageous because the sample represented various developmental delays among children assessed and treated in clinical settings. Fourth, the controls did not receive the same developmental tests as those received by children with developmental delays; therefore, we could not provide descriptive evaluation data, as provided for the children with developmental delays, to prove that the controls were true controls. Fifth, although all children with developmental delays were diagnosed using standardised developmental tests, we did not use molecular biomarkers to define the children’s diseases or characterise their symptom severity. The measurement of levels of neurotrophins, at least NGF and BDNF, in urine is necessary for enhancing the validity and generalisability of the current findings in the future. Sixth, the children could not completely define their health status because they were very young, had underdeveloped communication skills, or lacked concentration to assess their own views. Although we cannot rule out some probable differences between the children’s and parents’ perspectives about health, we used highly valid and reliable parent-reported health measurements for children. Seventh, HRQOL data were missing for dietary interventions. In addition to genetic polymorphisms, a folate-rich diet might affect the arsenic methylation capacity. Hence, data on the amount of folate or other vitamins and drugs that were consumed during the study might have affected the arsenic methylation capacity. Further information related to diet, nutrition, and drugs is required in future studies. Finally, the sample size was fairly small; a community-based longitudinal study with a larger sample size is required in the future for generalising the results.

## Conclusions

The present study confirmed that poor arsenic methylation capacity is associated with developmental delays in a dose-dependent manner in a sample larger than that of previous study. According to our review of relevant literature, this study is the first to demonstrate that poor arsenic methylation capacity (high MMA^V^ and low DMA^V^) is negatively associated with the health status, namely the HRQOL and functional performance, of children, particularly those with developmental delays.

## Methods

This prospective study was conducted at Shin Kong Wu Ho-Su Memorial Teaching Hospital in Taipei, Taiwan. Children aged 2–10 years with a diagnosis of developmental delays in fine motor, gross motor, cognition, social and emotional, and speech and language domains were eligible for this study. A developmental delay was defined as according to a performance 2 or more standard deviations lower than the mean on age-appropriate, standardised, norm-referenced tests. The assessment tools used included the Bayley III Scale of Infant and Toddler Development, Preschool Language Evaluation Tool, Child Expression Evaluation Tool, Chinese Wechsler Intelligence Scale for Children (3^rd^ edition), Gross Motor Function Measure, and Peabody Developmental Motor Scales. The family structure, the social support system, and hearing and visual acuity were routinely evaluated. Additional electroencephalography, cranial sonography, magnetic resonance imaging, and a genetic chromosomal check-up were conducted according to the children’s conditions. The detailed procedures are presented in our previous studies^16^,^17^. In total, 120 children with a diagnosis of developmental delays at the early developmental intervention clinic of the hospital were included, along with their mothers. In addition, 120 age- and sex-matched children who visited the paediatric clinics of the hospital for regular developmental evaluation and did not receive a diagnosis of developmental delays during the same period were recruited as the controls, along with their mothers.

The study was approved by the Institutional Review Board of Shin Kong Wu Ho-Su Memorial Hospital, in accordance with the World Medical Association Declaration of Helsinki (ClinicalTrials.gov: NCT02523989, date of registration: Aug 13, 2015). Informed written consent for the study was obtained from all of the parents of the children, and they granted permission for their and the children’s recruitment. A well-trained examiner administered structured questionnaires to the parents for assessing the health status of the children and its effect on their family function.

### Health status: HRQOL and functional performance of children

The HRQOL was measured using the parent-reported format of the PedsQL Generic Core Scales^44^. Higher scores represented a higher HRQOL. The Chinese version of the aforementioned scales yields satisfactory, feasible, reliable, and valid results^45^,^46^. The PODCI was used to assess the functional performance of the children by using a parent-reported form^47^, with scores ranging from 0 to 100–100 indicates the highest level of functional performance. The reliability of the Chinese version of the PODCI is satisfactory^46^,^48^.

### Family functioning: HRQOL, family influence, and psychological distress of parents

The Chinese version of the World Health Organization Quality of Life: Brief Version questionnaire was used to assess the parental HRQOL^49^. Scores range from 0 to 100, with a higher score indicating a higher HRQOL. This questionnaire has satisfactory reliability^46^,^49^. The PedsQL Family Impact Module was used to assess the effect of the children’s chronic conditions on the functioning of their parents and family^44^. Higher scores indicate a lower effect on the family. Moreover, the Chinese version of this module has satisfactory reliability^46^. The Hospital Anxiety and Depression Scale was used to measure parental psychological distress^50^; its Chinese version has satisfactory reliability^46^,^48^.

### Urine sample collection and analysis

Spot urine samples were collected from each participant, immediately frozen, and stored at −20 °C. In all urine samples, the levels of 4 forms of arsenic, As^III^, As^V^, MMA^V^, and DMA^V^, were measured through high-performance liquid chromatography (Waters 501; Waters Associates, Milford, MA, USA) combined with hydride generation atomic absorption spectrometry (Perkin–Elmer Flow Injection Analysis System 400-AA 100; Perkin–Elmer, Waltham, MA, USA). The detailed protocol was presented in our previous study^31^. The recovery rates of As^III^, As^V^, MMA^V^, and DMA^V^ ranged from 93.8% to 102.2%, with detection limits of 0.02, 0.07, 0.05, and 0.08 μg/L, respectively. For assessing the validity of the measurements, we purchased freeze-dried SRM 2670 urine, containing 480 ± 100 μg/L arsenic, from the National Institute of Standards and Technology (Gaithersburg, MD, USA). We detected arsenic at 507 ± 17 μg/L in the SRM 2670 standard (n = 4). For stabilising the urinary arsenic profiles, analyses were performed within 6 months of sample collection^51^.

### Statistical analysis

The results are expressed as the mean ± standard deviation. Chi-squared and t tests were used for analysing the demographic data of both study groups. All urinary arsenic profiles were normalised using urinary creatinine (μg/g or mg/g creatinine). The total urinary arsenic concentration (μg/g creatinine) was defined as the sum of As^III^, As^V^, MMA^V^, and DMA^V^ concentrations. The relative proportion of each arsenic species (InAs%, MMA^V^%, and DMA^V^%) was calculated by dividing the species concentrations by the total arsenic concentration. Multivariate logistic regression models were used to estimate odds ratios (ORs) and 95% CIs for determining the association between the urinary arsenic profile and developmental delay risk. Furthermore, multiple linear regression analyses were performed to identify the association between the arsenic methylation capacity and the health status of the children. Cut-off points for the values of various arsenic indices were the respective tertiles of the controls for dose–response analyses. Significance tests for the linear trend among ORs across exposure strata were performed by categorising exposure variables and considering scored variables as continuous. The potential confounding factors, namely age, sex, birth weight of the children, gestational weeks of their mothers, and urine creatinine, were adjusted in the multivariate models. All data were analysed using the Statistical Analysis Software package (version 9.1.2; SAS, Cary, NC, USA). The statistical significance level was set at a 2-sided *P* value of <05. The sample size was calculated assuming a standardised effect (mean difference and standard deviation) of 0.5. To achieve 95% power at a significance level of.05, the number of participants in each group had to be at least 110^16^.

## Additional Information

**How to cite this article**: Hsueh, Y.-M. *et al.* Association of Arsenic Methylation Capacity with Developmental Delays and Health Status in Children: A Prospective Case–Control Trial. *Sci. Rep.* **6**, 37287; doi: 10.1038/srep37287 (2016).

**Publisher’s note:** Springer Nature remains neutral with regard to jurisdictional claims in published maps and institutional affiliations.

## Figures and Tables

**Table 1:** Sociodemographic Characteristics of Children and Their Mothers.

Variables	Children with developmental delays (n = 120)	Children without developmental delays (n = 120)	P Value
Children			
Age (years)	5.62 ± 0.19	6.20 ± 0.25	0.07
BMI (kg/m^2^)	16.00 ± 0.30	16.59 ± 0.31	0.18
Birth weight (g)	2965.30 ± 58.55	3088.40 ± 51.14	0.11
No. of gestational weeks	37.56 ± 0.33	38.34 ± 0.22	0.05
Sex			
Male	68 (56.67)	68 (56.67)	1.00
Female	52 (43.33)	52 (43.33)	
Mothers			
Age (years)	35.72 ± 0.50	36.35 ± 0.48	0.36
BMI (kg/m^2^)	22.80 ± 0.40	22.52 ± 0.35	0.60
Gestational age (years)	30.02 ± 0.52	30.10 ± 0.44	0.90
Parity			
One	58 (49.57)	74 (61.67)	0.06
Two	43 (36.75)	39 (32.50)	
Three or more	16 (13.68)	7 (5.83)	
Educational level			
High school or lower	59 (49.17)	40 (33.33)	0.01
College or higher	61 (50.83)	80 (66.67)	

Values expressed as number (percent) or mean ± standard error unless noted otherwise.

BMI: body mass index.

**Table 2:** Health Status of Children and Their Family Impact.

Variables	Children with developmental delays		Children without developmental delays		*P* value
	N	Mean ± SE	N	Mean ± SE	
Health status of children					
PedsQL Generic Core Scales					
Physical health	120	74.35 ± 2.10	120	93.78 ± 1.04	<0.01
Psychosocial health	120	68.70 ± 1.56	120	85.96 ± 1.41	<0.01
Total health	120	69.91 ± 1.58	120	87.98 ± 1.24	<0.01
PODCI					
Upper extremity and physical functioning	110	84.55 ± 1.81	115	95.18 ± 0.83	<0.01
Transfer and basic mobility	110	93.12 ± 1.58	115	98.52 ± 0.43	<0.01
Sports and physical functioning	110	88.45 ± 1.64	115	95.83 ± 0.91	<0.01
Pain and comfort	110	84.97 ± 1.31	115	86.63 ± 1.30	0.37
Happiness	110	89.83 ± 1.13	115	78.91 ± 1.29	<.01
Global functioning	110	86.35 ± 1.13	115	92.70 ± 0.64	<0.01
Family function					
WHOQOL-BREF					
Physical health	118	44.16 ± 0.92	116	47.25 ± 0.99	0.02
Psychological	118	48.90 ± 1.14	116	51.77 ± 1.16	0.08
Social relationships	118	56.72 ± 1.28	116	62.54 ± 1.36	<0.01
Environment	118	52.19 ± 1.26	116	57.58 ± 1.21	<.01
PedsQL Family Impact Module					
Parent	120	65.97 ± 1.79	120	78.26 ± 1.65	<0.01
Family	120	62.61 ± 2.06	120	75.80 ± 1.92	<0.01
Total	120	64.98 ± 1.66	120	77.15 ± 1.60	<0.01
HADS					
Anxiety	120	6.74 ± 0.34	118	5.50 ± 0.35	0.01
Normal		74 (61.67)		95 (80.51)	<0.01
Abnormal		46 (38.33)		23 (19.49)	
Depression	120	7.03 ± 0.35	118	5.17 ± 0.34	<0.01
Normal		69 (57.50)		85 (72.03)	0.02
Abnormal		51 (42.50)		33 (27.97)	

PedsQL: Pediatric Quality of Life Inventory; PODCI: Pediatric Outcomes Data Collection Instrument; WHOQOL-BREF: World Health Organization Quality of Life: Brief Version; HADS: Hospital Anxiety and Depression Scale.

**Table 3:** Dose–Response Associations Between Urinary Arsenic Indices and Developmental Delay Risk in Children.

Variables	Children with developmental delays	Children without developmental delays	Multivariate ORs (95% CI)^a^
Urinary total arsenic (μg/L)	22.41 ± 1.68	16.87 ± 1.91	1.02 (1.00–1.03)
≤5.88	20 (16.67)	40 (33.33)	1.00^a,§^
5.88–17.78	40 (33.33)	40 (33.33)	2.21 (1.06–4.63)^*^
>17.78	60 (50.00)	40 (33.33)	3.75 (1.58–8.90)^**^
InAs%	5.99 ± 0.40	5.55 ± 0.61	1.02 (0.97–1.07)
≤2.32	27 (22.50)	40 (33.33)	1.00^b,§^
2.32–5.44	39 (32.50)	40 (33.33)	1.47 (0.75–2.89)
>5.44	54 (45.00)	40 (33.33)	2.12 (1.09–4.13)
MMA^V^%	3.67 ± 0.33	2.49 ± 0.33	1.10 (1.02–1.19)^*^
≤0.48	25 (20.83)	40 (33.33)	1.00^b,§^
0.48–2.14	34 (28.33)	40 (33.33)	1.37 (0.69–2.75)
>2.14	61 (50.83)	40 (33.33)	2.35 (1.22–4.53)^*^
DMA^V^%	90.34 ± 0.59	91.96 ± 0.78	0.97 (0.94–1.01)^+^
≤92.22	66 (55.00)	40 (33.33)	1.00^b,§^
92.22–96.07	25 (20.83)	40 (33.33)	0.37 (0.19–0.72)^**^
>96.07	29 (24.17)	40 (33.33)	0.41 (0.21–0.78)^**^

Values expressed as number (percent) or mean ± standard error unless noted otherwise.

^a^Adjusted for age, sex, the mother’s educational level, and urine creatinine level.

^b^Adjusted for age, sex, and the mother’s educational level.

Urinary total arsenic (μg/L): iAs^III^ + iAs^V^ + MMA + DMA.

InAs% = inorganic arsenic (iAs^III^ + iAs^V^)/total arsenic × 100.

MMA^V^% = MMA/total arsenic × 100.

DMA^V^% = DMA/total arsenic × 100.

^+^0.05 ≤ *P* < 0.1; ^*^P < 0.05; ^**^P < 0.01; ^§^*P* < 0.05 for trend test.

CI: Confidence interval; DMA^V^%: Dimethylasinic acid percentage; InAs%: Inorganic arsenic percentage; MMA^V^%: Monomethylarsonic acid percentage; OR: Odds ratio.

**Table 4:** Multiple Linear Regression Analyses of Arsenic Methylation Capacity and Health Status of Children.

Variables	All children		Children with developmental delays		Children with typical development	
	MMA^V^%^a^	DMA^V^%^a^	MMA^V^%^a^	DMA^V^%^a^	MMA^V^%^a^	DMA^V^%^a^
PedsQL						
Physical health	−1.46 (0.36)^**^	0.31 (0.18)^+^	−1.88 (0.59)^**^	0.63 (0.34)^+^	−0.29 (0.30)	−0.08 (0.13)
Psychosocial health	−1.19 (0.32)^**^	0.35 (0.16)^*^	−0.72 (0.45)	0.28 (0.25)	−0.82 (0.39)^*^	0.17 (0.17)
Total health	−1.24 (0.31)^**^	0.33 (0.16)^*^	−1.08 (0.45)^*^	0.39 (0.26)	−0.59 (0.35)^+^	0.09 (0.15)
PODCI						
Upper extremity and physical functioning	−1.14 (0.26)^**^	0.39 (0.13)^**^	−1.96 (0.47)^**^	0.82 (0.28)^**^	−0.30 (0.21)	0.12 (0.09)
Transfer and basic mobility	−0.88 (0.22)^**^	0.21 (0.11)^*^	−1.71 (0.44)^**^	0.55 (0.27)^*^	−0.02 (0.11)	0.02 (0.05)
Sports and physical functioning	−0.94 (0.26)^**^	0.23 (0.13)^*^	−1.39 (0.47)^**^	0.52 (0.27)^+^	−0.28 (0.24)	0.06 (0.10)
Pain and comfort	−0.91 (0.25)^**^	0.36 (0.12)^**^	−0.95 (0.37)^*^	0.45 (0.21)^*^	−0.67 (0.35)^+^	0.27 (0.15)^+^
Happiness	−0.82 (0.26)^**^	0.27 (0.13)^*^	−0.73 (0.38)^*^	0.48 (0.22)^*^	−0.59 (0.32)^+^	0.09 (0.13)
Global functioning	−0.89 (0.18)^**^	0.27 (0.09)^**^	−1.20 (0.32)^**^	0.50 (0.19)^**^	−0.39 (0.17)^*^	0.11 (0.07)

Values expressed as β (SE).

^a^Adjusted for age, sex, and the mother’s educational level.

^+^0.05 ≤ *P* < 0.1; ^*^P < 0.05; ^**^P < 0.01.

β: regression coefficient; PedsQL: Pediatric Quality of Life Inventory; PODCI: Pediatric Outcomes Data Collection Instrument. SE: Standard error.

## References

[b1] Rodríguez-Barranco M. *et al.* Association of arsenic, cadmium and manganese exposure with neurodevelopment and behavioural disorders in children: a systematic review and meta-analysis. Sci Total Environ 454–455, 562–577 (2013). 10.1016/j.scitotenv.2013.03.047 23570911

[b2] Falck A. J. *et al.* Developmental Exposure to Environmental Toxicants. Pediatr Clin North Am 62, 1173–1197 (2015). 26318946 10.1016/j.pcl.2015.05.005

[b3] Agency for Toxic Substances and Disease Registry (ATSDR). Toxicological profile for Lead. Atlanta, GA: US. Department of Health and Human Services, Public Health Service (2007).

[b4] Garcia-Vargas G. G. & Cebrian M. E. Health effects of arsenic. In: Wang L. W. , ed. Toxicology of metals. Boca Raton : CRC Press 423–438 (1996).

[b5] Basu A. *et al.* Creatinine, diet, micronutrients, and arsenic methylation in West Bengal, India. Environ Health Perspect 119, 1308–1313 (2011). 21652291 10.1289/ehp.1003393 PMC3230402

[b6] Yu H., Liu S., Li M. & Wu B. Influence of diet, vitamin, tea, trace elements and exogenous antioxidants on arsenic metabolism and toxicity. Environ Geochem Health 38, 339–351 (2016). 26169729 10.1007/s10653-015-9742-8

[b7] Abdul K. S., Jayasinghe S. S., Chandana E. P., Jayasumana C. & De Silva P. M. Arsenic and human health effects: A review. Environ Toxicol Pharmacol 40, 828–846 (2015). 26476885 10.1016/j.etap.2015.09.016

[b8] Dhar P., Mohari N. & Mehra R. D. Preliminary morphological and morphometric study of rat cerebellum following sodium arsenite exposure during rapid brain growth (RBG) period. Toxicology 234 **(1–2)**, 10–20 (2007). 17374429 10.1016/j.tox.2007.01.024

[b9] Kordas K. *et al.* Low-level arsenic exposure: Nutritional and dietary predictors in first-grade Uruguayan children. Environ Res 147, 16–23 (2016). 26828624 10.1016/j.envres.2016.01.022 PMC4821778

[b10] Bothwell M. NGF, BDNF, NT3, and NT4. Handb Exp Pharmacol 220, 3–15 (2014). 24668467 10.1007/978-3-642-45106-5_1

[b11] Ascano M., Bodmer D. & Kuruvilla R. Endocytic trafficking of neurotrophins in neural development. Trends in Cell Biology 22, 266–273 (2012). 22444728 10.1016/j.tcb.2012.02.005 PMC3348440

[b12] Ding Y. Q. *et al.* ProBDNF inhibits collective migration and chemotaxis of rat Schwann cells. Tissue Cell 48, 503–510 (2016). 27503312 10.1016/j.tice.2016.07.002

[b13] Katrin Deinhardt & Moses V. Chao. I. *Intercellular Signaling in Development and Disease: Cell Signaling Collection* how to reference books (ed. Katrin D.) 303–307 (Elsevier’s, 2011).

[b14] Park H. & Poo M. M. Neurotrophin regulation of neural circuit development and function. Nat Rev Neurosci 14, 7–23 (2013). 23254191 10.1038/nrn3379

[b15] Lou Y. *et al.* [Inhibitory effects of recombinant human neurotrophin-4/5 protein on neurotoxicity caused by arsenic trioxide]. Zhonghua Yu Fang Yi Xue Za Zhi. 33, 295–297 (1999). 11864496

[b16] Hsieh R. L. *et al.* Arsenic methylation capacity and developmental delay in preschool children in Taiwan. Int J Hyg Environ Health 217, 678–686 (2014). 24698386 10.1016/j.ijheh.2014.02.004

[b17] Hsieh R. L. *et al.* Quality of life and impact of children with unclassified developmental delays. J Paediatr Child Health. 49, E116–E121 (2013). 23316900 10.1111/jpc.12081

[b18] Ghosh A., Majumder S., Awal M. A. & Rao D. R. Arsenic exposure to dairy cows in Bangladesh. Arch Environ Contam Toxicol 64, 151–159 (2013). 23052359 10.1007/s00244-012-9810-3

[b19] Cui J., Shi J., Jiang G. & Jing C. Arsenic levels and speciation from ingestion exposures to biomarkers in Shanxi, China: implications for human health. Environ Sci Technol 47, 5419–5424 (2013). 23600923 10.1021/es400129s

[b20] Mandal B. K., Ogra Y., Anzai K. & Suzuki K. T. Speciation of arsenic in biological samples. Toxicol Appl Pharmacol 198, 307–318 (2004). 15276410 10.1016/j.taap.2003.10.030

[b21] Tsuji J. S., Garry M. R., Perez V. & Chang E. T. Low-level arsenic exposure and developmental neurotoxicity in children: A systematic review and risk assessment. Toxicology 337, 91–107 (2015). 26388044 10.1016/j.tox.2015.09.002

[b22] Howe C. G. *et al.* Folate and cobalamin modify associations between S-adenosylmethionine and methylated arsenic metabolites in arsenic-exposed Bangladeshi adults. J Nutr. 144, 690–697 (2014). 24598884 10.3945/jn.113.188789 PMC3985826

[b23] Paul S. & Giri A. K. Epimutagenesis: A prospective mechanism to remediate arsenic-induced toxicity. Environ Int 81, 8–17 (2015). 25898228 10.1016/j.envint.2015.04.002

[b24] Huang Y. K. *et al.* Plasma folate level, urinary arsenic methylation profiles, and urothelial carcinoma susceptibility. Food Chem Toxicol 46, 929–938 (2008). 18054417 10.1016/j.fct.2007.10.017

[b25] Chung C. J. *et al.* Polymorphisms in one-carbon metabolism pathway genes, urinary arsenic profile, and urothelial carcinoma. Cancer Causes Control 21, 1605–1613 (2010). 20532609 10.1007/s10552-010-9589-3

[b26] Shen H. *et al.* Factors Affecting Arsenic Methylation in Arsenic-Exposed Humans: A Systematic Review and Meta-Analysis. Int J Environ Res Public Health. 13, 205 (2016). 26861378 10.3390/ijerph13020205 PMC4772225

[b27] Jain R. B. Association of arsenic exposure with smoking, alcohol, and caffeine consumption: data from NHANES 2005-2010. Environ Toxicol Pharmacol. 39, 651–658 (2015). 25682010 10.1016/j.etap.2015.01.011

[b28] Ma M. & Le X. C. Effect of arsenosugar ingestion on urinary arsenic speciation. Clin Chem. 44, 539–550 (1998). 9510859

[b29] Steinmaus C., Yuan Y., Kalman D., Atallah R. & Smith A. H. Intraindividual variability in arsenic methylation in a U.S. population. Cancer Epidemiol Biomarkers Prev 14, 919–924 (2005). 15824164 10.1158/1055-9965.EPI-04-0277

[b30] Concha G., Vogler G., Nermell B. & Vahter M. Intra-individual variation in the metabolism of inorganic arsenic. Int Arch Occup Environ Health 75, 576–580 (2002). 12373320 10.1007/s00420-002-0361-1

[b31] Hsueh Y. M. *et al.* Urinary levels of inorganic and organic arsenic metabolites among residents in an arseniasis-hyperendemic area in Taiwan. J Toxicol Environ Health A 54, 431–444 (1998). 9661909 10.1080/009841098158728

[b32] Kaya-Akyüzlü D., Kayaaltı Z., Doğan D. & Söylemezoğlu T. Does maternal MDR1 C1236T polymorphism have an effect on placental arsenic levels? Environ Toxicol Pharmacol. 41, 142–146 (2016). 26694653 10.1016/j.etap.2015.11.019

[b33] Punshon T. *et al.* Placental arsenic concentrations in relation to both maternal and infant biomarkers of exposure in a US cohort. J Expo Sci Environ Epidemiol. 25, 599–603 (2015). 25805251 10.1038/jes.2015.16 PMC4583336

[b34] Vahter M. Effects of arsenic on maternal and fetal health. Annu Rev Nutr. 29, 381–399, doi: 10.1146/annurev-nutr-080508-141102 (2009). 19575603

[b35] Chen Y. *et al.* Arsenic exposure from drinking water, arsenic methylation capacity, and carotid intima-media thickness in Bangladesh. Am J Epidemiol. 178, 372–381 (2013). 23788675 10.1093/aje/kwt001 PMC3727341

[b36] Dhar P., Mohari N. & Mehra R. D. Preliminary morphological and morphometric study of rat cerebellum following sodium arsenite exposure during rapid brain growth (RBG) period. Toxicology 234, 10–20 (2007). 17374429 10.1016/j.tox.2007.01.024

[b37] García-Medina N. E. *et al.* Conditioned flavor aversion and brain Fos expression following exposure to arsenic. Toxicology 235, 73–82 (2007). 17420081 10.1016/j.tox.2007.03.009 PMC1924883

[b38] Gumilar F., Lencinas I., Bras C., Giannuzzi L. & Minetti A. Locomotor activity and sensory-motor developmental alterations in rat offspring exposed to arsenic prenatally and via lactation. Neurotoxicol Teratol 49, 1–9 (2015). 25725132 10.1016/j.ntt.2015.02.005

[b39] Mundey *et al.* Antioxidant Potential of Ocimum sanctum in Arsenic Induced Nervous Tissue Damage. Braz J. Vet. Pathol 6, 95–101 (2013).

[b40] Gardner R. M., Kippler M., Tofail F., Bottai M., Hamadani J., Grandér M. *et al.* Environmental exposure to metals and children’s growth to age 5 years: a prospective cohort study. Am J Epidemiol 177, 1356–1367 (2013). 23676282 10.1093/aje/kws437 PMC3676155

[b41] Nadeau K. C. *et al.* In utero arsenic exposure and fetal immune repertoire in a US pregnancy cohort. Clin Immunol 155, 188–197 (2014). 25229165 10.1016/j.clim.2014.09.004 PMC4309995

[b42] Ahmed S. *et al.* Arsenic exposure and cell-mediated immunity in pre-school children in rural Bangladesh. Toxicol Sci 141, 166–175 (2014). 24924402 10.1093/toxsci/kfu113 PMC4833103

[b43] Edwards M., Hall J., Gong G. & O’Bryant S. E. Arsenic exposure, AS3MT polymorphism, and neuropsychological functioning among rural dwelling adults and elders: a cross-sectional study. Environmental Health 13, 15 (2014). 24621105 10.1186/1476-069X-13-15 PMC4234288

[b44] Varni J. W., Seid M. & Rode C. A. The PedsQL: measurement model for the pediatric quality of life inventory. Med. Care 37, 126–139 (1999). 10024117 10.1097/00005650-199902000-00003

[b45] Chan L. F., Chow S. M. & Lo S. K. Preliminary validation of the Chinese version of the Pediatric Quality of Life Inventory. Int. J. Rehabil. Res. 28, 219–227 (2005). 16046915 10.1097/00004356-200509000-00004

[b46] Hsieh R. L., Huang H. Y. & Lee W. C. The Correlation of Pediatric Outcome Data Collection Instrument with Health Related Quality of Life, Emotion in Children and Their Parents with Developmental Delays. Taipei: Taiwan Academy of Physical Medicine and Rehabilitation (2009).

[b47] Haynes R. J. & Sullivan E. The Pediatric Orthopaedic Society of North America pediatric orthopaedic functional health questionnaire: an analysis of normals. J Pediatr Orthop 21, 619–621 (2001). 11521030

[b48] Hsieh R. L., Lin M. I., Huang H. Y. & Lee W. C. The relationship between pediatric outcomes data collection instruments and functional impairment in developmentally delayed Chinese children and their parents’ health: implications for child and family-centered medicine. Int J Pers Cent Med 1, 1–8 (2011).

[b49] Hwang H. F., Liang W. M., Chiu Y. N. & Lin M. R. Suitability of the WHOQOL-BREF for community-dwelling older people in Taiwan. Age. Ageing 32, 593–600 (2003). 14599999 10.1093/ageing/afg102

[b50] Zigmond A. S. & Snaith R. P. The hospital anxiety and depression scale. Acta Psychiatr Scand 67, 361–370 (1983). 6880820 10.1111/j.1600-0447.1983.tb09716.x

[b51] Chen Y. C., Amarasiriwardena C. J., Hsueh Y. M. & Christiani D. C. Stability of arsenic species and insoluble arsenic in human urine. Cancer Epidemiol Biomarkers Prev 11, 1427–1433 (2002). 12433722

